# Clinical Remarks upon Surgical Cases in the Buffalo General Hospital—Operations for Extraction of Cataract

**Published:** 1868-12

**Authors:** J. F. Miner


ART. II.—*Clinical Remarks upon Surgical Cases in the Buffalo General Hospital—Operations for Extraction of Cataract.* BY J. F. MINER, M. D.

GENTLEMEN:—I regret these cases of cataract could not be presented before you at a later period of the term, after the whole subject had been fully considered in the lecture room. I will, however, briefly state some of the general facts connected with this disease, and the principles upon which the various operations for its removal are based.

Cataract is opacity of the crystaline lens, or of the capsule of the lens, or of both of these combined, and may be *congenital*—appearing at, or soon after birth; *idiopathic* or primary disease; or *traumatic*, that is, arising from injury. In congenital cataract the lens is soft; in traumatic cataract also, the lens is soft, that is, it has at least its usual semi-gelatinous consistency, and can be easily cut through with any fine needle or instrument, and is called soft to distinguish it from that condition assumed by the lens in age, or in many instances of cataract appearing in persons past the middle period of life, called hard cataract. We have then, *hard* and *soft* cataract; the methods of distinguishing between them will be fully described hereafter. The causes of congenital cataract are not very apparent, but it seems peculiar to some families, all the children perhaps having cataract at, or soon after birth. The disease as it appears in persons past the middle period of life can generally be traced to no plain or obvious cause. Punctured wounds of the globe, especially if the capsule of the lens is at all disturbed, are almost certain to produce opacity; blows upon the temple or head, and particularly blows upon the globe of the eye cause cataract. The causes of cataract, then, are constitutional or general, and local, the first of which are not much understood, while the latter are sufficiently obvious. The diagnosis cannot be difficult; all you have to do is to place your patient in good light and standing directly in front, you will see behind the pupil, the milky white lens. You can hardly mistake it for any other condition of the eye, even in its early stages, it will be distinctly visible, and all refinement of examination to determine its character is quite unnecessary; reflections from mirrors or other sources are to avoided, and your diagnosis is not very liable to be incorrect. You have only to observe these patients before operation and you will ever after readily recognize the disease.

Medicine has no influence upon the progress or termination of cataract, and you will never prescribe drugs either for its prevention or cure. Charlatans have sometimes practiced upon the credulous—have deceived them by dilating the pupil with belladonna or atropia, its active principle, and thus admitting more light into the eye have temporarily improved vision, but beyond this, no improvement can be made in vision by the use of medicine.

All operations for its cure are comprised in *two* or perhaps *three* general plans. The lens can be removed from the eye by different modes of operation; it can be displaced and removed from the field of vision; and, it can be divided and its capsule ruptured, the aqueous humor is thus admitted to its substance by which it is dissolved, or as it is called, absorbed.

Soft cataract requires for its removal rupture of the capsule, or rupture of capsule and division of the lens; the process of removal than proceeds from natural causes. Hard cataract may be displaced below the axis of vision or extracted from the eye. Displaced, it is liable to cause inflammation in the choroid retina or iris—to act something like a foreign body, and by its presence finally induce changes in these delicate structures, which are fatal to vision. Generally, however, the lens is mostly dissolved or absorbed, even if quite hard, and thus good results are often obtained by the operation by *reclination* or *couching*, as it is called. This plan of operation has been extensively practiced, and has afforded on the whole very favorable results, but in hard cataract it is not the best operation which it is possible to make, and at the present time the best operators never adopt it. The less experienced choose it, since it is vastly easier of execution, and exposes the surgeon and perhaps the patient to fewer risks. Only the easy and expert operator should attempt operation by extraction, the plan by reclination or couching is undoubtedly safest and best with inexperienced surgeons, while extraction offers advantages when it is skillfully made.

Please observe the preparation and mode of making operation for hard cataract. Our first patient, Miss West, has had the lens very successfully removed from the right eye, by the same method, in May last. She has now returned for a similar operation upon the other eye. No general preparation of the system is necessary; she was directed not to take breakfast this morning, hoping thus to avoid vomiting from the chloroform, and the iris has been dilated with atropine. When *completely* under the influence of chloroform and not until the anæsthesia is complete, the cataract knife is made to enter the anterior chamber of the eye; to pass rapidly and steadily through it, and thus to make section of the upper third of the cornea near its union with the sclerotic. The manner of this section is one of the important steps in the operation and attention is to be directed to it. The knife is to be passed through this chamber so steadily and quickly that the aqueous humor does not escape until the section is *nearly* complete, otherwise the iris may be protruded before the knife and embarrass the procedure. When the opening in the cornea has been made, and the water in the chamber has escaped, the next step necessary, is to rupture the capsule of the lens, which is done with a cataract needle or other sharp instrument introduced through the corneal wound and passed through the pupil to the lens; when this is completed the opake body often immediately presents itself at the corneal opening, and with very little assistance makes its escape; in this instance it is so, and I pass the lens to you for examination. The upper eyelid is now raised and the cornea adjusted with care, so that there may be early union. The lid is drawn carefully over the wound, a graduated compress placed over the eye, and roller bandage applied to afford pressure and support to the globe. The removal of the lens in this instance has been entirely satisfactory and no accident of any kind has embarrassed the procedure. So far as can be judged, the highest expectation of its success may be indulged; but there are yet sources of danger and failure which no operative skill can remove; these will be fully explained to you hereafter.

Our second patient is in all respects so far as important clinical facts are concerned a repetition of the first. She is 45 years old, with cataract fully formed in both eyes. She is therefore practically blind, and the results of our operation are as important to her almost as life itself. The same preparation by dilating pupil, giving chloroform, etc., etc., has been made, and the same operation practiced. In one eye the lens did not escape so entire and unbroken as in the other; it was soft and less easily removed, and the operation was prolonged and embarrassed by it, but it is by no means certain that it will not afford equally as good vision.

You have this morning had opportunity to observe the operation for extraction of cataract in three eyes, but I regret it could not have been afforded you after having fully studied the subject. There are several other modes by which the diseased lens is removed from the field of vision, and it would have been instructive to have varied our process to these various ways which surgeons have adopted for this purpose. My sense of duty to my patients has alone prevented it, and I have chosen the one which my experience and judgment dictate as the best. It has been proposed of late years to make iridectomy—section of the iris—previous to, or in connection with, this mode of removing the lens, the idea being that the lens would escape easier after a part of the iris had been removed. It appears to me wholly unnecessary in most cases, as the lens will pass readily through the pupil when it is dilated without any such section. A part of the iris can be removed with great safety, but it is a deformity to the eye, and as a rule, is wholly unnecessary. There may be cases where such preliminary or accompanying operation is desirable, but I am convinced that it ought not to be made for the purpose of facilitating the escape of the lens, when the eye retains its normal conditions in other respects. This field is too extensive for even a notice of the important questions involved, and I must defer further comments for future opportunity.

